# A Rare Case and Literature Review of Pyelo-Hepatic Abscess in an Immunocompetent Patient: When Effective Source Control and Targeted Antimicrobial Therapy Might Not Be Enough

**DOI:** 10.3390/microorganisms12101989

**Published:** 2024-09-30

**Authors:** Anita Sforza, Andrea Bonito, Giorgio Tiecco, Giovanni Moioli, Samuele Storti, Marco Lechiara, Francesco Castelli, Eugenia Quiros-Roldan

**Affiliations:** 1 Department of Clinical and Experimental Sciences, Unit of Infectious and Tropical Diseases, University of Brescia, ASST Spedali Civili, 25123 Brescia, Italy; anisforza@gmail.com (A.S.); g.tiecco@unibs.it (G.T.); s.storti@unibs.it (S.S.); francesco.castelli@unibs.it (F.C.); 2 Operating Unit of Infectious and Tropical Diseases, ASST Spedali Civili, 25123 Brescia, Italy; andreabonito@hotmail.com (A.B.); giovanni.moioli@alice.it (G.M.); 3 Unit of Diagnostic Radiology 1, ASST Spedali Civili, 25123 Brescia, Italy; marco.lechiara@libero.it

**Keywords:** pyelo-hepatic, abscess, urinary stent, Candida, *Escherichia coli*, TZP-R/Ceph3-S

## Abstract

Pyelo-hepatic abscess is a rare complication of upper urinary tract infections (UTIs). We describe a case of polymicrobial pyelo-hepatic abscess in an immunocompetent patient. A 71-year-old male patient with a double-J stent for right ureteral lithiasis was admitted in our Infectious Diseases Department for a pyelo-hepatic abscess. Despite a targeted antibiotic therapy against an extended spectrum betalactamase-negative *Escherichia coli*, the patient did not improve. Further examinations revealed a possible polymicrobial aetiology, including *Candida* spp. and *E. coli* resistant to piperacillin/tazobactam but sensitive to third-generation cephalosporins. To date, a paucity of articles regarding pyelo-hepatic abscess exist, consisting mostly of case reports. Urinary stones and a ureteral stent indwelling time exceeding 90 days are known risk factors for upper UTIs and for bacterial dissemination in contiguous organs. Pyelo-hepatic abscesses usually involve Gram-negative bacilli, but they can be polymicrobial, including fungi. As a range of factors could limit the efficacy of antibiotics inside an encapsulated lesion and might contribute to the selection of resistant species during treatment, clinicians should be aware of this complication and try to prevent this event by acting on the main modifiable risk factor.

## 1. Introduction

Pyelo-hepatic abscess is a rare complication that could develop from an upper urinary tract infection (UTI) when conditions that increase pressure, such as urinary stones or benign prostatic hypertrophy (BPH), lead to the spread of the infection beyond the renal capsule in the surrounding organs [1]. The microorganisms most involved in upper UTIs are Gram-negative bacilli (especially *Escherichia coli*), but in men with urological pathologies, non-*E. coli* Gram-negative bacilli, Gram-positive bacteria and *Candida* spp. are more frequent, and even a polymicrobial involvement is possible [2,3]. Ureteral stents are a known risk factor for UTIs. Colonisation rates are high, but only a minority of patients develop a symptomatic UTI [4]. The incidence increases proportionally to the indwelling time and with systemic diseases, such as diabetic nephropathy or chronic kidney disease [4]. Various factors can lead to a reduction in antibiotic penetration into an encapsulated purulent lesion and, therefore, limit its effectiveness [5]. This is why, in the presence of large encapsulated lesions, a combined medical and surgical therapy is often necessary to promote healing, reduce the duration of treatment and avoid the development of drug resistance [6].

Here, we describe a rare case of polymicrobial pyelo-hepatic abscess in an immunocompetent patient with an initially unfavourable evolution despite an effective source control and targeted antimicrobial therapy.

## 2. Case Report

**Figure 1:**
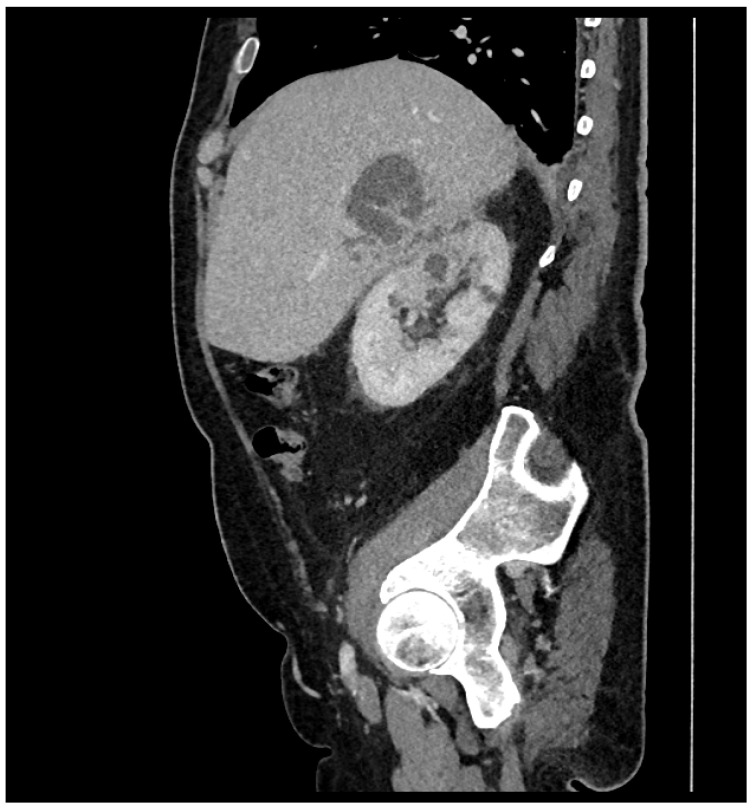
Abdomen CT scan. Sagittal section of the first abdominal CT scan performed showing an abscessualised right pyelonephritis and an intra-hepatic abscess.

In May 2022, a 71-year-old male patient was admitted to our Infectious Diseases Department for lumbar and abdominal pain, dysuria, fever (started 15 days earlier) and anaemia. He reported the following comorbidities on admission: diabetes mellitus (DM) and BPH. Multiple transurethral resections of a bladder tumour were performed, and a double-J stent was recently placed for a right ureteral lithiasis and hydroureteronephrosis. Increased inflammatory markers and nitrites in the urine were found. Empirical antibiotic therapy with intravenous piperacillin/tazobactam (TZP) 4.5 g every 6 h was started based on a clinical suspicion of pyelonephritis and the potential risk of healthcare-associated infection. An abdominal CT scan showed a renal abscess in communication with a purulent hepatic fluid collection (Figure 1) on which a percutaneous drainage was performed on day 1.

A TZP-susceptible and extended spectrum betalactamase (ESBL)-negative *E. coli* was found in both urine and fluid drainage cultures (Table 1), but blood cultures were negative.

**Figure 2:**
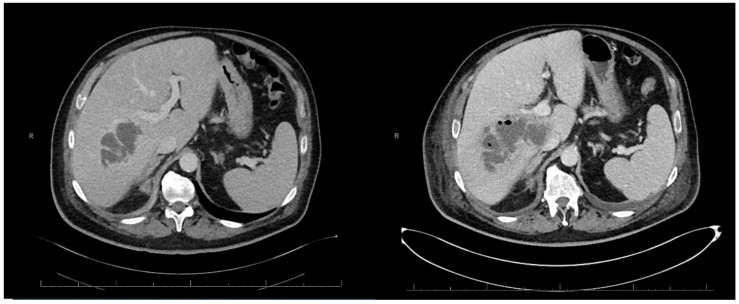
Abdomen CT scan. Temporal evolution of the intra-hepatic abscess visible in two axial sections of the first abdominal CT scans (on the **left**) and the second (on the **right**) taken 7 days apart.

On day 6, despite targeted antibiotic therapy with intravenous TZP 4.5 g every 6 h, the patient did not improve, and a double-J stent replacement was performed. On day 7, a follow-up abdominal CT scan showed an increase in the intra-hepatic abscess (Figure 2).

Consequently, a liver drainage was placed, and a new collection of purulent fluid was performed. No additional invasive surgical interventions were considered by the surgical specialists at our hospital. On day 8, the patient experienced a clinical worsening with desaturation and hypertensive crisis. The XR scan showed a voluminous right pleural effusion requiring diuretic and oxygen therapy. As the removed double-J stent culture was positive for fluconazole-sensitive *Candida albicans* (Table 2), intravenous fluconazole 400 mg/die was started.

The PCR result for *C. albicans* on the blood culture was negative, and no *C. albicans* grew from the liver drainage liquid. On day 10, an ESBL-negative *E. coli* was isolated from the second liver drainage liquid culture; however, this time, it was highlighted to be resistant to TZP (Table 1). Antibiotic therapy was escalated by switching TZP to intravenous cefepime 2 g every 8 h. In the following days, a gradual clinical improvement was recorded: the patient was apyretic and reported a significant decrease in abdominal pain, so the liver drainage was removed. On day 14, the patient underwent an evacuative thoracentesis, and considering the respiratory dynamic improvement, oxygen therapy was discontinued. Culture tests and a PCR for *C. albicans* performed on the pleural fluid were negative. On day 26, a CT scan of the abdomen showed a significant decrease in the intra-hepatic abscess size. Clinical conditions improved, and a progressive decline in the inflammatory markers was observed. On day 35, the double-J stent and the ureteral stone were surgically removed. Intravenous antimicrobial therapy with cefepime and fluconazole was continued until the discharge. On day 42, the patient was discharged, antimicrobial therapy was switched to oral administration with cefixime 400 mg every 8 h and fluconazole 400 mg every 24 h. The patient presented to our outpatient clinic two weeks following the completion of antimicrobial therapy. He reported no clinical symptoms, and laboratory results were within normal ranges. Follow-up ultrasonography demonstrated complete resolution of the pyelo-hepatic abscess.

## 3. Discussion

To date, the literature on pyelo-hepatic abscesses consists mostly of case reports. We reviewed the current literature [7], including all articles published in peer-reviewed medical journals, regarding pyelo-hepatic abscesses. We excluded articles published in non-English languages, pre-print or ahead of print analyses, pre-clinical studies, reviews, systematic reviews and metanalyses. We consulted the United States National Library of Medicine, PubMed, the Cochrane Library, MedlinePlus and NLM Gateway (last accessed September 2024). References were identified with the following research term combinations: “abscess” AND “pyelohepatic” OR “pyelo-hepatic” OR “pyelo hepatic” OR “nephron-hepatic” OR “renalhepatic” OR “renal-hepatic” OR “renal hepatic”. A total of nine articles were assessed for eligibility, but six were excluded as they did not report cases of pyelo-hepatic abscess. A team of two resident doctors in Infectious and Tropical Diseases of the University of Brescia, Italy, independently read the abstract and full text of the two included scientific works (AS, GT) [8,9,10], and an Infectious and Tropical Diseases Specialist of the ASST Spedali Civili of Brescia, Italy, revised both the included and excluded papers (AB). All information collected in the selected articles is summarised in Table 3.

In all of the included articles, the patients involved had a right urinary tract lithiasis. In one of them [10], the patient underwent a partial hepatectomy and a right nephrectomy, so the clinicians were able to diagnose a xanthogranulomatous pyelonephritis (XGPN), which is usually associated with nephrolithiasis and could cause an infiltration into surrounding organs [11]. We could not confirm or rule out XGPN in our patient, although in the literature, other cases of XGPN complicated by hepatic extension are described [10,11]. In the literature, only one case of pyelo-hepatic abscess in a female patient has been reported [8], which may be attributed to the hepatic extension of an inadequately treated renal bacterial infection [8]. Indeed, urinary stones are a known risk factor for upper UTIs and for dissemination of infection into contiguous organs by increasing the pressure inside the urinary tract [1]. A diagnostic delay could also facilitate the spread of microorganisms: our patient had been symptomatic for 15 days before being admitted to the hospital. He had various risk factors for both UTIs and the spreading of a renal abscess: a ureteral stent for ureteral lithiasis, DM and BPH. Similarly, Tanwar et al. described a pyelo-hepatic abscess complicated by a right pleural effusion [8]. Pleural effusion can be a frequent consequence of hepatic abscess when inflammatory involvement of the diaphragm can cause increased permeability of the lymphatic vessels and thus a build-up of fluid in the diaphragmatic pleura [12].

Regarding ureteral stents, colonisation rates are between 42 and 90% [4]. Bacteria can interact with the stent surface, and the presence of a urinary conditioning film raises bacterial adhesion and the formation of the biofilm [4]. Despite the high colonisation rate, only a small number of patients develop a symptomatic UTI [4]. Indwelling time exceeding 90 days and systemic diseases are associated with a higher risk of UTIs, so the shortening of indwelling time, especially for patients with risk factors, could be an implementable strategy to prevent these complications [13].

In our case, we found a polymicrobial involvement of the urinary tract. Renal and perinephric abscesses are usually due to Gram-negative enteric bacilli, but they could have a polymicrobial aetiology [3]. In different observational retrospective studies, polymicrobial abscesses have been reported with a frequency that varies from 2 to 33% [14]. Fungi like *Candida* spp. are often involved, mainly in male patients with systemic pathologies like DM, a history of urological pathologies or risk factors for immunosuppression [2,14]. Hepatic abscesses are usually polymicrobial, with a fungal component that has been reported in 22% of liver abscesses [15]. *Candida* spp. is the leading cause of fungal liver infection in immunocompromised patients but, in approximately 2% of cases, could also be found in liver abscesses of immunocompetent patients [16]. However, we could not isolate *Candida* spp. in any of the cultures performed on the liver abscess drainage liquid. Given the incidence of polymicrobial infections, a broader-spectrum empirical antimicrobial therapy may be warranted in cases involving encapsulated lesions.

Usually, the treatment of superficial abscesses consists of a combination of antibiotics and percutaneous drainage [6]. However, although consensus is lacking, antimicrobial therapy alone can be successful on small and multiple abscesses [6]. In a literature review of 465 medically treated abscesses, a success rate of antimicrobial therapy of 85,9% was reported. The main factor associated with unsuccessful medical treatment was a diameter greater than or equal to 5 cm [17]. Similarly, in a retrospective study, Hope et al. reported a 100% success rate with antibiotic therapy alone for unilocular hepatic abscess < 3 cm in 107 patients [18]. The penetration of antibiotics into an abscess depends on several features: the permeability of an encapsulated lesion (influenced by the duration of infection and the stage of maturation), the pH gradient and the abscess surface area-to-volume ratio [5]. High concentration levels of antibiotics in an abscess are reached later than plasma peak levels; this is believed to make the activity of “time-dependent” antibiotics more effective than that of “concentration-dependent” ones [5]. However, there are other factors that could limit antibiotic efficacy, for example, acid pH and low oxygen, high levels of protein and bacterial count, presence of enzymes that inhibit antimicrobial agents and sequestration of bacteria in leukocytes [5].

In the first culture performed on the drainage liquid, we isolated an *E. coli* susceptible to both TZP and third-generation cephalosporins (Ceph3), while the second one was resistant to TZP. In accordance with current guidelines and Italian epidemiological data, piperacillin/tazobactam was selected due to the suspicion of a healthcare-associated infection, primarily driven by the presence of an indwelling device. Comorbidities such as diabetes and BPH have influenced our choice of treatment, although they are not currently established risk factors for pyelo-hepatic abscesses. However, the large use of TZP has increased the diffusion of resistant species [19]. The same resistance mechanisms used by bacteria against TZP are also responsible for the resistance to many subclasses of β-lactam antibiotics, such as Ceph3, so *Enterobacteriaceae* that are resistant to TZP are usually also resistant to Ceph3 [20]. However, there is a phenotype of *Klebsiella pneumoniae* and *E. coli* that is TZP resistant but susceptible to third-generation cephalosporins (TZP-R/Ceph3-S) due to a different resistance mechanism that is believed to be the hyperproduction of β-lactamase TEM-1 (blaTEM-1) and SHV-1 [21]. Tazobactam can inhibit the activity of class A β-lactamases; therefore, the presence of blaTEM-1 should not give resistance to TZP. However, two studies have linked amplification and hyperproduction of blaTEM-1 with this phenotype [22,23]. A precedent exposure to β-lactam/β-lactamase inhibitors has been reported to be a risk factor associated with the TZP-R/Ceph3-S phenotype [24].

All the discussed factors limiting the efficacy of antibiotics inside an encapsulated lesion might have contributed to the selection of resistant species like the TZP-R/Ceph3-S *E. coli* described in our case.

## 4. Conclusions

In patients with known risk factors, such as ureteral lithiasis, DM, BPH and a double-J stent, it is important to consider pyelo-hepatic abscesses as a possible, although rare, evolution of an upper UTI. Clinicians should be aware of this complication and try to prevent this event by acting on the main modifiable risk factor and shortening the timing of ureteral stent indwelling. These abscesses are usually due to Gram-negative bacilli but might also have a polymicrobial aetiology, even in non-immunosuppressed patients. The different pharmacological properties of antibiotics in an encapsulated lesion could lead to the exposure of microorganisms at sub-inhibitory concentrations and, therefore, the selection of resistant microbial species such as TZP-R/Ceph3-S *Enterobacteriaceae*. On small and multiple abscesses, it seems that medical therapy alone is sufficient, but on the management of large abscesses, the antibiotic therapy is often not enough, and percutaneous drainage is unavoidable.

## Figures and Tables

**Table 1:** *E. coli* antibiograms (S = susceptible, R = resistant, NT = not tested, TZP-S/Ceph3-S = susceptible to piperacillin/tazobactam and third-generation cephalosporines, TZP-R/Ceph3-S = resistant to piperacillin/tazobactam but susceptible to third-generation cephalosporines.

	*Escherichia coli* TZP-S/Ceph3-S		*Escherichia coli* TZP-S/Ceph3-S		*Escherichia coli* TZP-R/Ceph3-S	
	Uroculture (Day 1)		Liver Drainage (Day 1)		Liver Drainage (Day 7)	
		MIC (mcg/mL)		MIC (mcg/mL)		MIC (mcg/mL)
Amikacin	S	2	S	2	S	2
Amoxicillin/clavulanic acid	R	>16	R	>16	R	>16
Cefepime	S	≤0.12	S	≤0.12	S	≤0.12
Cefotaxime	S	≤0.25	S	≤0.25	S	≤0.25
Ceftazidime	S	0.25	S	0.25	S	0.25
Ceftazidime/avibactam	NT	NT	S	≤0.12	S	0.25
Ceftolozane/tazobactam	NT	NT	S	≤0.25	S	≤0.25
Ciprofloxacin	I	0.5	I	0,5	R	1
Ertapenem	S	≤0.12	NT	NT	NT	NT
Fosfomycin	S	≤16	NT	NT	NT	NT
Gentamicin	S	≤1	S	≤1	S	≤1
Imipenem	S	≤0.25	S	≤0.25	S	≤0.25
Meropenem	S	≤0.25	S	≤0.25	S	≤0.25
Nitrofurantoin	S	≤16	NT		NT	
Piperacillin/tazobactam	S	≤4	S	≤4	R	64
Trimethoprim/sulfamethoxazole	S	≤20	S	≤20	S	≤20

**Table 2:** *C. albicans* antibiogram. (S = susceptible, R = resistant, NT = not tested, NR = not reported).

*Candida albicans*		
Double-J Stent Culture		
		MIC (mcg/mL)
Amphotericin B	S	0.5
Anidulafungin	S	0.03
Caspofungin	S	NR
Fluconazole	S	0.25
Isavuconazole	NR	≤0.008
Itraconazole	S	0.06
Micafungin	S	0.015
Posaconazole	S	0.03
Voriconazole	S	≤0.008

**Table 3:** Summary table regarding study characteristics, aetiologic data, microbiological sample and causes of pyelo-hepatic abscess (Tanwar R, *Ind. J. Urol.* 2013; Chung SD, *Urol.*, 2008).

Authors	Journal	Year	Article	Aetiology	Sample	Cause of Pyelo-Hepatic Abscess
[8]	*J. Med. Case Rep.*	2023	Case Report	*Proteus mirabilis*	Urine culture	Extension of a renal infectious focus
[9]	*Ind. J. Urol.*	2013	Case report	*Escherichia coli*	Blood culture	Dissemination of pyonephrosis due to nephrolithiasis
[10]	*Urol.*	2008	Case report	*Proteus mirabilis*	Urine and drainage culture	Xanthogranulomatous pyelonephritis with nephrolithiasis

## Data Availability

The datasets generated during and analysed during the current study are not publicly available due to privacy reasons but are available from the corresponding author on reasonable request.
